# Recommendations for the development, implementation, and evaluation of technology-assisted physical activity interventions to support healthy aging: insights from PhysAgeNet

**DOI:** 10.3389/fdgth.2026.1913687

**Published:** 2026-09-28

**Authors:** Nadja Schott, Jana Pelclová, Eleftheria Giannouli, Antoine Langeard, Christopher McCrum, Rosemary Dubbeldam, Rafael A. Bernardes, Arben Boshnjaku, David Beckwée

**Affiliations:** 1 Department of Psychology and Human Movement Science, Institute for Sport and Movement Science, University of Stuttgart, Stuttgart, Germany; 2 Faculty of Physical Culture, Palacký University Olomouc, Olomouc, Czechia; 3 Rehabilitation Engineering Laboratory, Department of Health Sciences and Technology, ETH Zurich, Zurich, Switzerland; 4 COMETE U1075, Université de Caen Normandie, INSERM, CYCERON, Caen, France; 5 Department of Nutrition and Movement Sciences, Institute of Nutrition and Translational Research in Metabolism (NUTRIM), Maastricht University, Maastricht, Netherlands; 6 Department of Movement Science, Institute of Sport and Exercise Sciences, University of Münster, Münster, Germany; 7 Nursing Research Innovation and Development Centre of Lisbon (CIDNUR), School of Nursing of the University of Lisbon (ESEUL), Lisbon, Portugal; 8 Faculty of Medicine, University “Fehmi Agani” in Gjakova, Gjakovë, Kosovo; 9 Rehabilitation Research Group, Department of Physiotherapy, Human Physiology and Anatomy, Vrije Universiteit Brussel, Jette, Belgium; 10 Research Group MOVANT, Department of Rehabilitation Sciences and Physiotherapy, Universiteit Antwerpen, Wilrijk, Belgium

**Keywords:** behavior change, digital health, FITT-VP principles, healthy aging, older adults, physical activity, technology acceptance, technology-assisted interventions

## Abstract

Digital health technologies offer substantial potential to support physical activity among older adults, yet their development and implementation often remain driven by technological possibilities rather than clinical evidence and user needs. This position paper, developed within the PhysAgeNet COST Action, synthesizes current evidence and expert consensus to provide recommendations for designing, evaluating, and implementing technology-assisted physical activity interventions for older adults. We first outline key technology categories and functions relevant to physical activity promotion, including wearables, exergames, mobile applications, telehealth platforms, and smart environments, and highlight critical technical requirements such as accuracy, reliability, connectivity, interoperability, and data security. We then emphasize the need to align digital interventions with established clinical guidelines for physical activity, falls prevention, frailty, and sarcopenia, including explicit specification of exercise dose according to FITT-VP principles and robust monitoring of dual adherence to both physical activity and technology use. Building on technology acceptance models and behavior change theories, we describe how user-centered and co-creative design processes can translate theoretical constructs into concrete digital features that enhance motivation, enjoyment, and long-term engagement. Finally, we propose stakeholder-specific guidance for developers, researchers, and clinicians, including recommendations on user assessment, recruitment of vulnerable groups, implementation frameworks, and clinically grounded evaluation of outcomes and usability. Together, these recommendations provide a pragmatic roadmap for moving from a technology-push paradigm toward evidence-based, acceptable, and equitable technology-assisted physical activity interventions that can be sustainably integrated into routine practice for older adults.

## 1 Introduction

Digital health technologies have opened new opportunities to promote physical activity (PA) among older adults. These innovations represent a powerful technology push, yet adoption remains uneven. *The technology push* describes innovations driven by technological capability rather than user need. Developers create devices and applications first, then seek clinical applications, often misaligning with older adults’ capabilities, clinicians’ requirements, and evidence-based guidelines. As a result, their adoption and sustained use can lag behind the pace of innovation.

A central challenge lies in bridging two worlds: the technology sector and real-world implementation. Developers often emphasize technical capabilities, while clinicians focus on evidence, guidelines, and patient outcomes. These divergent priorities impede the translation of promising digital tools into routine practice. Building a shared vocabulary and fostering mutual understanding are essential to ensure that new technologies are both backed by solid scientific evidence and practical to use in real-world settings.

The PhysAgeNet COST Action network (4 workgroups; https://physagenet.eu/) was established to address specific challenges related to evidence-based PA interventions in old age, including those involving technology (1). Working Group 3's core role was to map and critique how technology-assisted exercise trials in older adults are currently done and to turn this into concrete, consensus-based methodological guidelines for future research and technology development. In this context, the current paper presents a position statement that articulates a shared vision for the future development, research, and practical use of technology-assisted PA interventions for older adults. By integrating insights from technology, health sciences, and real-world practice, it aims to guide researchers, developers, and practitioners toward co-created solutions that are evidence-based, acceptable, and impactful.

While this paper builds on and synthesizes recent PhysAgeNet outputs (2–5), its distinct contribution is to integrate these previously separate strands (technology categorization, clinical dosing standards, technical requirements, behavior-change theory, and co-design methods) into a single, stakeholder-specific set of actionable recommendations, and to introduce and operationalize the dual adherence construct (Section 3, Section 7.1) as an organizing principle that has not been articulated in this form in the prior network outputs. Throughout this paper, “older adults” refers to individuals aged 65 years and above, consistent with the age threshold applied in Table 1; we note that this definition varies across the cited guidelines and evidence base, and that chronological age is an imperfect proxy for the functional and cognitive heterogeneity that shapes technology use in this population.

**Table 1:** Structured clinical assessment framework for technology-assisted physical activity interventions in older adults, outlining key domains, clinical aims, and concrete examples across pre-intervention, peri-intervention, and post-intervention phases.

Aspect	Aim & considerations	Examples
**PRE-INTERVENTION**		
Eligibility criteria	Define inclusion based on intrinsic capacities (e.g., locomotor, sensory, cognitive), contextual factors, safety, and feasibility rather than age alone.	-For walking intervention: age ≥65 years and able to walk 10 m independently (with or without aid).
		-Visual acuity sufficient to read 18-point font on a tablet screen.
		-Home environment allows ≥2 m^2^ of safe exercise space.
Safety screening	Ensure medical safety and identify exercise contraindications.	-Physical Activity Readiness Questionnaire for Everyone (PAR-Q+) completed before enrolment (88).
		-Review of fall history (≥2 falls in last 6 months → supervised format).
Motivation	Identifies sources of enjoyment and motivational regulation to tailor behavioral support.	-Behavioral Regulation in Exercise Questionnaire (BREQ-3) to identify intrinsic vs. controlled motivation (89).
		-Physical Activity Enjoyment Scale (PACES): enjoyment profile (90).
Digital Literacy	Assess the ability to operate basic digital tools required for the intervention.	-Practical task: download an app, log in, adjust volume.
		-Mobile Device Proficiency Questionnaire MDPQ score (91).
		-Check the ability to charge and sync the wearable device independently.
Technology acceptance	Identify perceived usefulness, ease of use, and potential anxiety.	-Senior Technology Acceptance Model (STAM) baseline score (92).
		-Technology Readiness Index (TRI) for optimism, innovativeness, discomfort and insecurity (93).
		-Question: “What concerns do you have about using this device?”
Sensory/cognitive constraints	Adapt the interface and supervision level to impairments.	-Mini-Cog or Montreal Cognitive Assessment (MoCA-Blind) (https://mocacognition.com).
		-Snellen chart for vision.
		-Whisper test for hearing.
		-Adjust tablet font ≥18–20 pt if mild visual impairment.
Contextual & socio-economic feasibility	Ensure real-world practicality of the intervention.	-Access to smartphone/tablet & stable internet.
		-Financial feasibility (device subscription costs).
		-Language proficiency.
		-Health literacy considerations.
Clinical decision implications (summary step)	Integrate findings to determine intervention format.	-Define supervision level (unsupervised/hybrid/supervised).
		-Prescribe intensity based on functional tests.
		-Decide on the technology complexity level.
		-Determine need for caregiver involvement
**PERI-INTERVENTION**		
Technology onboarding and training	Build confidence and reduce technology-related anxiety	-60-minute onboarding session (1:1 or small group).
		-Step-by-step printed guide with screenshots.
		-Practice session, including starting/stopping session independently.
Ongoing technical support	Prevent dropout due to technical barriers.	-Dedicated phone number available during office hours.
		-Weekly proactive check-in call during the first month.
		-In-app troubleshooting FAQ with short videos
Social and informal support	Enhance adherence by involving caregivers and families.	-Invite a family member to the onboarding session.
		-Provide caregiver quick-start sheet.
		-Monthly “bring-a-relative” group session.
Adaptation and personalization	Adjust intensity, content, and interface based on progress and tolerance.	-Automatic progression when participant completes ≥80% sessions/week.
		-Reduce intensity if heart rate >85% age-predicted max repeatedly.
		-Switch from text to audio prompts if reading difficulty is observed.
Monitoring, safety, and optimization	Ensure safety and refine intervention delivery.	-App usage logs (sessions/week).
		-Weekly RPE (Borg scale) entry (94).
		-Short 5-min phone check every 2 weeks.
		-Flag inactivity >7 days → follow-up call.
**POST-INTERVENTION**		
Physical outcomes and safety	Evaluate functional change and adverse events.	-Timed Up and Go test (TUG) pre-post comparison (95).
		-30-second Chair Stand Test (96).
		-Handgrip dynamometry (kg) (97).
		-6-Minute Walk Test (distance change ≥30 m considered meaningful) (98).
		-Adverse event log review.
Adherence (PA and technology)	Distinguish between exercise adherence and technology use adherence.	-App-based participation frequency.
		-Device usage logs.
		-Self-reported barriers.
Psychological and motivational outcomes	Assess sustainability potential.	-Repeat PACES and BREQ-3.
		-10-minute semi-structured interview: “Would you continue this program? Why/why not?”
Technology usability and acceptability	Evaluate experienced usability after real-world use.	-System Usability Scale (SUS) score (≥70 acceptable) (99).
		-Post-intervention STAM comparison.
		-Open-ended question on burden or frustration.
Global perceived impact	Capture broader life impact and autonomy.	-Likert-scale question: “I feel more confident moving independently” (1–5).
		-Short qualitative interview on autonomy, confidence, and participation in daily life.

The following sections provide concise guidance for key stakeholders, covering an overview of technologies and key definitions, the integration of evidence-based guidelines into digital solutions, and essential technological factors such as accuracy, connectivity, interface design, security, and system integration. They also address determinants of technology acceptance and sustained use, the roles of behavior change theory and user-centered design, and specific considerations for developers, researchers, and clinicians. Together, these sections form a roadmap to ensure that digital innovations translate into meaningful, equitable, and evidence-based improvements in the health and well-being of older adults.

## 2 Technologies to support physical activity

The rapid digitalization of health has profoundly shaped how physical activity programs are designed and delivered. Over the past decade, the term mobile health (mHealth) has largely been replaced by the broader concept of digital health, encompassing the Internet of Things (IoT), smart devices, big data, and related innovations. Digital health can be defined as the integration of digital technologies into health-related processes to enhance their effectiveness and efficiency. Technologies ranging from pedometers to telehealth platforms are increasingly used to support physical activity in older adults, with emerging, though still heterogeneous, evidence that they may enhance engagement and accessibility (2, 6). Table 2 provides an overview of the most common technologies related to physical activity, their definitions, and examples.

**Table 2:** Overview of the most common technologies that may support physical activity [see also (2)].

Technology	Definition	Example
Wearables/Trackers	Body-worn electronic devices (e.g., smartwatches, fitness trackers) that monitor and record physiological or activity data with real-time feedback capabilities.	Fitbit, Apple Watch, Garmin, Oura
Sound and video recording systems	Audio and video capture systems for demonstrating exercises (pre-recorded) and providing feedback (live recording) during physical activity sessions.	Voice devices, video recording platforms, webcams
Apps	Software applications for mobile devices, tablets, or web platforms that deliver exercise programs, track activity, and facilitate remote coaching.	Web-based apps, mobile smartphone apps, and telepresence programs
Skeleton avatar systems	Camera-based motion capture systems using artificial intelligence to track body movements and create virtual skeletal models for movement analysis.	Kinect-based exergames (e.g., YourShape)
Multi-sensor setups	Integrated sensor arrays combining accelerometers, gyroscopes, barometers, and location sensors (e.g., BLE, UWB) for precise activity monitoring.	IMU wearables, multi-sensor activity monitors
Smart fitness equipment	Exercise equipment with integrated sensors and connectivity providing interactive training with real-time feedback for strength, cardio, or resistance exercises.	Peloton Bike, step/dance mats, pressure platforms, digital elastic bands
Live Streaming and Online Fitness Classes	Video platforms delivering real-time or recorded group exercise sessions for remote participation with instructor interaction.	Zoom-based senior fitness classes; live streaming platforms
Interactive Games and Extended Reality: Exergames	Digital games combining physical activity with motion tracking and immersive environments (VR/AR) to enhance engagement through gamification.	Oculus Quest exergames; Meta-Quest, PlayStation VR fitness; Google glasses
Augmented reality	Wearable display technology that superimposes digital information onto the real world to provide visual cues, form feedback, and safety alerts during physical activities.	AR glasses (e.g., HoloLens), smartphone AR apps
Client Apps for Activity Tracking and Community Engagement	Mobile applications provide activity logging, goal-setting, social features, and motivational reminders to support adherence and community connection.	Strava, MyFitnessPal, Peloton, Freeletics, Noom
Telehealth and Remote Coaching Platforms	Digital platforms enable virtual consultations, personalized exercise prescription, supervised physical activity, and progress monitoring via video and data sharing.	Teladoc, Physera, PT Pal

A recent review found that Exergames (30%), mobile applications (22%), and web video/phone calls (13%) were the most commonly used digital tools, while virtual and augmented reality were used infrequently (2).

Technology has been incorporated into PA programs for several purposes: to enable independent and/or safer physical activity; provide information before, during, or after activity; monitor users and outcomes; individualize programs; and facilitate social support, including in home-based settings. Communication features can operationalize behavior change techniques (see Table 3), for example by providing exercise instructions, outcome monitoring (e.g., walking distance), feedback on exercise performance, real-time health or movement data, and information on functional ability (2).

**Table 3:** Overview of behavioral change models and integrative frameworks relevant to technology-assisted physical activity interventions in older adults.

Framework/Theory	Core principle	Relevance for technology-assisted PA in older adults (examples)	Reference
**Foundational motivational and social theories**			
Theory of Reasoned Action (TRA)	Attitudes, subjective norms → behavioral intention → behavior	Explains adoption of digital PA tools through perceived expectations of peers, family, and healthcare providers; highlights the role of social approval and normative influence beyond individual beliefs.	(28)
Theory of Planned Behavior (TPB)	Attitudes, subjective norms, perceived behavioral control → intention and behavior	Captures confidence in managing PA and using digital technologies; informs interventions improving PA adherence	(43)
Health Belief Model (HBM)	Perceived susceptibility, severity, benefits, barriers; cues to action; self-efficacy	Explains technology uptake by addressing perceived benefits and barriers (e.g., cost, usability, privacy)	(44)
Self-Regulation Theory (SRT)	Goal setting, self-monitoring, feedback, and behavioral adjustment	Supports wearables and digital tools for monitoring progress, feedback, and adaptive goal setting	(45)
Self-Determination Theory (SDT)	Autonomy, competence, relatedness	Apps can personalize goals, provide adaptive feedback, and offer peer communities.	(46)
Social Cognitive Theory (SCT)	Self-efficacy, modeling, social support	Digital features can enhance self-efficacy via feedback, group sessions, and peer modeling.	(47)
Transtheoretical Model (TTM)	Stages of change	Programs can tailor content to the readiness stage	(48)
Affective-Reflective Theory (ART)	Dual-process: impulsive affect + reflective evaluation	Explains the role of enjoyment in exergaming, highlights affective drivers	(49)
**Integrative frameworks**			
COM-B	Capability, Opportunity, Motivation → Behavior	Diagnostic model to identify barriers, guides design features	(50)
Health Action Process Approach (HAPA)	Motivation–volition, planning, coping	Supports action planning, coping strategies in digital interventions	(51)
**Design frameworks**			
Behavior Change Wheel (BCW)	COM-B linked to intervention functions and BCTs	Provides a stepwise structure to design digital features	(50)
Theoretical Domains Framework (TDF)	14 psychological domains	Helps identify barriers/enablers in tech adoption	(52)
Fogg Behavior Model (FBM)	Behavior = Motivation × Ability × Prompt	Highlights the importance of simplicity and timely prompts	(53)
Persuasive System Design (PSD)	Persuasive design principles	Guides the design of apps, wearables, and exergames	(54)
**General and contextual frameworks**			
PICOTS-ComTeC framework	Structured reporting across dimensions	Ensures systematic specification of digital interventions	(55)
Ecological models	Multi-level influences on behavior	Emphasize the role of social and environmental context	(56, 57)

## 3 Evidence-based clinical approaches

Technology-assisted PA programs must align with established guidelines such as those developed by the World Health Organization [WHO (7); see Table 4].

**Table 4:** Clinical guidelines that describe evidence-based physical activity recommendations in older people.

Clinical Guidelines	Reference
WHO 2020 guidelines on physical activity and sedentary behavior	(7)
EULAR recommendations for the non-pharmacological core management of hip and knee osteoarthritis: 2023 update	(8)
World guidelines for falls prevention and management for older adults: a global initiative.	(9)
Exercise and Sports Science Australia updated its position statement on exercise for preventing falls in older people living in the community	(10)
Evidence on physical activity and falls prevention for people aged 65 + years: systematic review to inform the WHO guidelines on physical activity and sedentary behavior	(11)
Resistance exercise as a treatment for sarcopenia: prescription and delivery	(12)
Screening, Diagnosis and Management of Sarcopenia and Frailty in Hospitalized Older Adults: Recommendations from the Australian and New Zealand Society for Sarcopenia and Frailty Research (ANZSSFR) Expert Working Group	(13)
Exercise to Prevent and Manage Frailty and Fragility Fractures	(14)
Physical Exercise as Therapy for Frailty	(15)
Evidence-based recommendations for resistance and power training to prevent frailty in community-dwellers	(16)
Clinical practice guidelines for the prevention and management of frailty: A systematic review	(17)
International Exercise Recommendations in Older Adults (ICFSR): Expert Consensus Guidelines	(18)
Global consensus on optimal exercise recommendations for enhancing healthy longevity in older adults (ICFSR)	(19)

Digital tools should deliver exercise content consistent with the best available evidence, following recognized standards for frequency, intensity, time, and type of activity. Adherence is a critical determinant of success in technology-assisted PA interventions. Because there is a clear dose-response relationship between PA and health outcomes in older adults generally (7), maintaining consistent participation in recommended exercise is essential. Adherence encompasses both completion of prescribed activities, defined according to the American College of Sport Medicine FITT-VP framework as frequency, intensity, time, type, volume, and progression (20), and sustained engagement with the technology that delivers or supports them (3). This dual adherence is vital: technology functions not only as a delivery tool (e.g., mobile apps or wearables), but also as a behavioral target that requires ongoing interaction. Ultimately, the extent to which users engage with both the physical activity and the technology determines the intervention's effectiveness. Policy and implementation strategies should therefore promote digital solutions that integrate clinical best practices while supporting sustained user engagement. To move this construct from a conceptual to a measurable framework, we propose that dual adherence be operationalized along two distinct but interacting dimensions. The first, activity-component adherence, captures the extent to which the prescribed FITT-VP parameters (Section 7.1) are actually completed, and can be quantified as the proportion of prescribed sessions completed, prescribed intensity or duration achieved (e.g., via heart-rate or accelerometer data against target zones), and progression adherence over the intervention period. The second, technology-engagement adherence, captures sustained interaction with the delivery platform itself, and can be quantified as login or session frequency relative to the prescribed schedule, feature-use breadth, and engagement decay over time. No universal validated thresholds can currently be recommended across technologies and intervention types; adequate thresholds should therefore be prespecified in relation to the prescribed activity dose and the intended use of the technology. Technology engagement should not be treated as a proxy for activity adherence. Depending on the intervention logic, it may act as a mediator or moderator of activity adherence and health outcomes, and these relationships should be tested explicitly when the study design and sample size permit. Reporting the two dimensions separately, rather than a single composite “adherence” figure, allows researchers to distinguish interventions that fail because users disengage from the technology from those that fail despite continued technology use but insufficient activity dosage. This distinction has direct implications for whether troubleshooting should target the exercise prescription, the platform design, or both. Table 5 (Section 7.1) illustrates how these two dimensions can be captured within a single reporting template alongside the FITT-VP prescription.

**Table 5:** Worked example: operationalizing FITT-VP prescription and dual adherence reporting for an illustrative walking intervention.

FITT-VP parameter	Illustrative prescribed value	Activity-component adherence metric	Technology-engagement adherence metric
Frequency	5 sessions/week	Proportion of prescribed sessions completed per week (app-logged)	Number of app opens per week vs. 5 prescribed reminders
Intensity	Moderate (40%–59% heart-rate reserve, or RPE 12–13/20)	Proportion of session time within target heart-rate zone (wearable data)	Use of real-time intensity feedback feature (yes/no per session)
Time	30 min/session	Mean session duration completed vs. 30 min prescribed	Time spent actively viewing/interacting with the app per session
Type	Brisk walking (aerobic)	Modality adherence: sessions performed as walking vs. substituted activity	Use of the activity-type logging feature
Volume	150 min/week	Weekly accumulated minutes vs. 150 min target	Weekly summary/dashboard views
Progression	+5 min/session every 2 weeks up to 45 min	Achieved progression vs. planned progression schedule	Engagement with progression prompts/notifications

## 4 Technical considerations

Technical considerations play a decisive role in the feasibility, effectiveness, and adoption of digital physical activity interventions. Table 6 summarizes the main technical considerations.

**Table 6:** Technical considerations (21–23).

Technical consideration	Description	Example
Accuracy	Precision of data measurement and validity compared to gold-standard methods	Validation against an appropri-ate criterion measure in the intended older-adult population, with error estimates reported.
Reliability	Consistency and reproducibility of measurements across time and conditions	Report test-retest agreement using appropriate metrics (e.g., intraclass correlation coeffi-cients with confidence intervals) in the intended population.
Internet Connectivity	Network infrastructure requirements for data synchronization and video-based interventions, with potential barriers in rural or low-resource settings	Interventions requiring video calls or cloud data syncing may not be feasible without consistent Wi-Fi or mobile data service
Power and Charging Requirements	Battery life limitations and user burden of maintaining device charge	Match charging frequency to the intervention context and report battery performance under realistic use conditions; provide reminders or support where needed.
Technical Support and Maintenance	Availability of assistance for installation, troubleshooting, and ongoing device management	Remote support systems, dedicated help lines, or caregiver training programs for device setup and maintenance
Interoperability	Ability of systems to communicate and share data across platforms, and minimum hardware/software specifications	Document the platform inter-faces and data standards used (e.g., Apple HealthKit, Android Health Connect, or standards-based APIs).
Data Security	Protection of personal health information during transmission, storage, and processing	End-to-end encryption of physiological data; GDPR compliance; secure cloud storage with multi-factor authentication
Integration with other (healthcare) systems	Compatibility with electronic health records and clinical decision support systems	Use clearly specified exchange standards and data structures (e.g., HL7 FHIR resources) for clinically relevant summaries.
Device Availability and Cost	Financial and logistical barriers to accessing required hardware (smartphones, wearables, sensors)	Provision of subsidized devices or loan programs for participants without personal technology
Wearable Compliance	Adherence to consistent device wear patterns is required for valid data collection	Non-compliance factors: forgetting to wear the device, skin irritation, charging burden, aesthetic concerns
Environmental Setup	Physical installation requirements for home-based monitoring equipment	Motion sensors, smart scales, or camera systems requiring caregiver or technician-assisted installation
Artificial intelligence integration	Application of machine learning algorithms for data analysis and personalized feedback with privacy protections	Report model performance to-gether with hardware and com-munication requirements, in-cluding battery drain, memory footprint, latency, and training or update time.

Several considerations in Table 6 merit further explanation. Beyond interface simplicity, technology-assisted PA interventions should be evaluated against formal software ergonomics standards. ISO 9241-11:2018 provides a framework for assessing usability in terms of effectiveness, efficiency, and satisfaction, while ISO 9241-171:2025 addresses software accessibility for users with diverse physical, sensory, and cognitive abilities (24, 25). Before clinical deployment, developers should document a staged quality-assurance process that includes formative usability testing with representative older adults, expert review against relevant human-factors criteria, testing of foreseeable failure scenarios (such as sensor dropout, connectivity loss, low battery, or incorrect device placement), and field validation under realistic conditions. Where the software is a medical device or an integral part of one, relevant medical-device software lifecycle requirements, such as IEC 62304, and applicable risk-management requirements should also be considered (26). The applicable standards and level of validation will depend on the intended use, risk profile, and regulatory context.

Hardware constraints also determine whether privacy-preserving artificial-intelligence approaches are feasible. Many consumer wearables have limited processing capacity, memory, battery life, and connectivity; continuous on-device training or frequent model updates may therefore increase energy and communication demands. Depending on the intervention, developers may consider lightweight on-device inference, periodic processing on a paired smartphone or home hub, and scheduling computationally intensive operations during charging or reliable connectivity. Federated- or split-learning approaches should therefore not be recommended solely on privacy grounds without also considering their computational, communication, and energy requirements on the intended hardware. Rather than prescribing a single architecture, studies should report the target hardware, sampling and transmission strategy, battery drain, memory footprint, latency, and model performance so that feasibility can be judged in the intended setting.

Interoperability requires more than a general statement of “FHIR compatibility.” At minimum, interoperability reporting should address four architectural domains: device or platform data capture, standardized data exchange, secure user authorization, and semantic consistency across systems. Depending on the context, possible components may include vendor or platform interfaces (e.g., Apple HealthKit or Android Health Connect), HL7 FHIR resources for data exchange, authorization and identity standards such as OAuth 2.0 and OpenID Connect, SMART on FHIR for standards-based application integration with electronic health records, and established clinical terminologies (27). The precise architecture will vary across countries and health systems; therefore, we recommend transparent reporting of the standards and interfaces used rather than a single universal blueprint.

The ageism-in-AI concerns discussed in Section 7.6 also have a data-governance dimension. In addition to standard ethical and legal requirements, consent information should be accessible to older users with varying digital literacy and should clearly explain which data are collected, how they are processed, who can access them, whether they may be used for algorithm development or secondary purposes, and how participants can exercise applicable data-protection rights. Where feasible, consent to participate should be separated from consent to optional data uses, such as long-term storage, algorithm development, secondary research, or third-party sharing, allowing users to grant or withhold permission for each purpose independently. Layered plain-language information, optional visual summaries, and comprehension checks, including teach-back where appropriate, may support understanding. Data minimization, purpose limitation, appropriate security controls, and transparent procedures for modifying or withdrawing consent should be adapted to the applicable legal and regulatory context. Involving older adults in the design of consent materials, consistent with the co-creative methodologies in Table 7, may improve their clarity and relevance.

## 5 Theoretical frameworks for technology-assisted PA interventions

In recent decades, scientists have used various models of health behavior to explain technology acceptance and use among older people (see Figures 1, 2). Most of these models are based on the established Theory of Reasoned Action (TRA) developed by Fishbein and Ajzen (28). This theory of human behavior assumes that actual behavior is preceded by an intention to behave in a certain way. This intention, in turn, is influenced by two factors: the attitude toward the behavior and the subjective norm. While the attitude toward the behavior depends on positive or negative beliefs and assumptions about the action and its outcome (evaluative affect), the subjective norm is based on normative assumptions (social desirability) and the motivation to live up to them.

**Figure 1:**
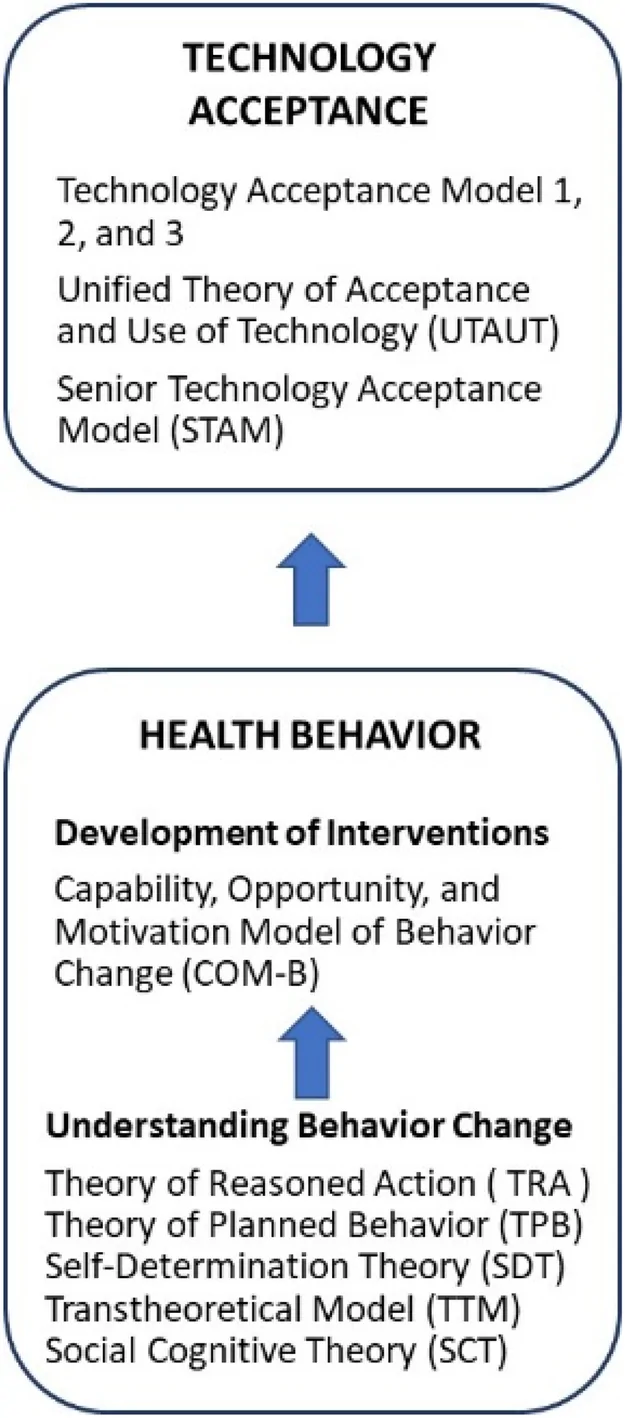
Overview of behavior change theories and technology acceptance models.

**Figure 2:**
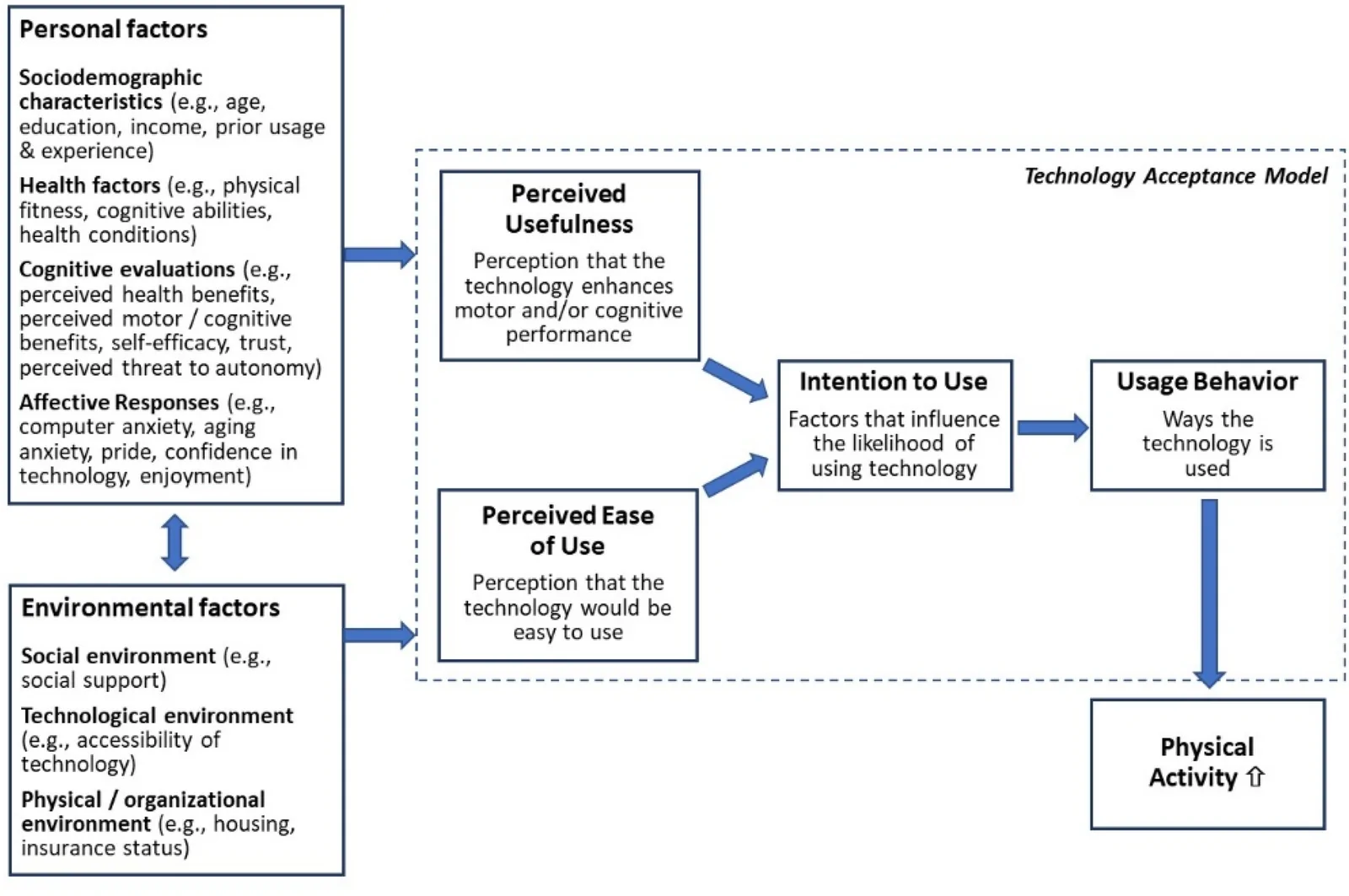
Using the technology acceptance model to identify factors that predict the likelihood of increased physical activity among older adults: personal and environmental factors influence perceived usefulness and ease of use (TAM), which shape attitudes and intentions, which, in turn, drive technology-based health behaviors. Extended TAM models in health explicitly integrate these personal (motivation, risk perception) and environmental (support, usability, policies) factors to better explain why people do or do not adopt health technologies. [based on concepts described in (29–31)].

Technology acceptance is typically defined as the behavioral intention to use or acquire knowledge of technology and can be regarded as a precursor to its practical implementation [technology adoption (32);], as well as to its favorable reception and ongoing use of newly introduced devices and systems (33). In recent decades, various models of health behavior have been used to explain technology acceptance and use among older people. Table 8 provides an overview of the most commonly used models.

While technology acceptance models and their senior-specific extensions explain why older adults adopt digital technologies, they provide limited insight into how sustained engagement in technology-assisted physical activity is achieved. Acceptance represents an important precondition, but long-term effectiveness depends on whether interventions successfully target the behavioral and motivational mechanisms underlying physical activity.

Accordingly, technology-assisted physical-activity and exercise interventions must be grounded in robust behavior-change theory to maximize adherence and effectiveness. This foundation spans (a) traditional models developed for face-to-face or community programs, (b) more integrative frameworks tailored to technology-mediated contexts, and (c) practical toolkits that translate theory into design features. In addition to these well-established models, emerging dual-process perspectives, such as the Affective-Reflective Theory, are increasingly recognized as important for understanding the emotional dimension of exercise engagement, particularly in contexts such as exergaming. Reflecting this evolution, a recent scoping review calls for an overarching, modular meta-framework that integrates shared constructs across theories and captures their dynamic influence over time (39).

Table 3 provides an overview of behavioral change models and integrative frameworks relevant to technology-assisted physical activity interventions in older adults. Although these frameworks differ in scope and level of abstraction, they converge in identifying a limited set of behavioral mechanisms that underpin successful intervention outcomes. Evidence syntheses consistently indicate that effective technology-assisted PA programs operationalize these mechanisms through a core set of behavior-change techniques, including self-monitoring, goal-setting, feedback, prompts or cues, and social support (40–42).

Table 3 catalogs these frameworks individually, but they are not interchangeable, and no single framework addresses all determinants of technology-assisted PA behavior. In practice, we suggest they are complementary at different stages of intervention design: the Behaviour Change Wheel and Social Cognitive Theory are best suited to specifying ‘active ingredients’ (behavior change techniques, self-efficacy-building strategies) during intervention design; the Technology Acceptance Model and UTAUT are best suited to explaining and predicting the technology-engagement dimension of dual adherence (Section 3) once a prototype exists to be evaluated; and socio-ecological models are best suited to identifying contextual barriers (e.g., social support, environmental access) during implementation and scale-up planning, rather than to specifying either the exercise prescription or the interface itself. A technology-assisted PA intervention is therefore unlikely to be adequately theorized by any one framework in isolation: we recommend that researchers explicitly pair a behavior-change or clinical framework addressing the activity-component adherence with a technology-acceptance framework addressing the technology-engagement component, and justify that pairing in relation to the intervention's stage of development, rather than citing multiple frameworks as parallel, undifferentiated justifications.

## 6 User-centered development methodologies

Development of technologies to support PA in older adults should incorporate co-creative processes involving experts from sports science, gerontology, human-computer interaction, and older adults themselves. Indeed, user-centered design is paramount when developing technology-based assessment tools for older adults. In addition, during development, it is important to test and evaluate prototypes iteratively to encourage feedback loops between users and developers. This is essential for refining technologies and ensuring they meet the needs of older adults. Table 7 provides an overview of methodologies that incorporate end-user involvement in technology development.

**Table 7:** Methodologies that incorporate end-user involvement during the development of technologies [for a review, see (58–60)].

User-centered development models	Core principle	Reference
Co-creative Interdisciplinary Exergame design (CIEM)	Interdisciplinary teams and end users iteratively design engaging exergames through workshops/prototypes.	(61, 102)
Co-creative Interdisciplinary Exergame Design Process Model-Extended Reflection (CIEMER)	CIEM extension: Adds reflection cycles for iterative refinement post-testing.	(61, 62)
Experience-Based Co-Design (EBCD)	Patients/clinicians co-create services through storytelling, touchpoints, and prototypes.	(63)
Participatory Action Research (PAR)	Cyclical: Plan-act-observe-reflect with older adults as co-researchers.	(64)
Intervention Mapping (IM) with Co-Creation	Stepwise: Needs assessment, objectives, and methods co-developed with end-users.	(65)

**Table 8:** Technology acceptance models.

Technology Acceptance Models	Description	Example	Reference
Technology Acceptance Model (TAM)	TAM-1:Core: Perceived Usefulness (PU: improves PA outcomes) and Perceived Ease of Use (PEOU: simple interface) predict intention and use.	Fitbit app for step tracking	(30)
	TAM-2: TAM + subjective norm, image, job relevance, output quality, result demonstrability.	Wearable feedback for social sharing	(34)
Unified Theory of Acceptance and Use of Technology (UTAUT)	Integrates 8 models: Performance Expectancy, Effort Expectancy, Social Influence, Facilitating Conditions, moderated by age/gender.	Group exergame classes via Zoom	(35)
	UTAUT-2: UTAUT + hedonic motivation, price value, habit; better for consumer contexts like apps.	Gamified PA app with rewards	(36)
Senior Technology Acceptance Model (STAM)	TAM extension for seniors: Adds resistance factors (e.g., cognitive decline, tech anxiety).	Senior-friendly tablet for balance exercises	(37)
Gerontechnology Acceptance Model	Holistic for aging: Tech anxiety, self-efficacy, compatibility with life values.	Voice-activated home gym coach	(38)

### 6.1 What aspects should technology developers consider?

Technology interventions should be designed with a user-centric approach to cater to the diverse needs of older adults (see also Table 7). This should follow an iterative approach, ensuring that feedback from older end users is continuously integrated across all stages of technology readiness, from initial ideation to final market deployment (66). Older adults often value simplicity and ease of use, as complexity can lead to frustration and non-adoption. For instance, the Digital Assessment of Precise Physical Activity (DAPPA) tool was developed using a person-based approach, ensuring it was easy to navigate and comprehensive in its reporting of activities (67). Similarly, the development of mobile applications for older adults emphasizes the importance of simplicity and intuitive navigation (68).

Interaction design is another essential consideration. Interfaces must be suited to the sensory and motor capabilities of older adults. Technologies that use large fonts, clear instructions, and multimodal feedback, such as visual and auditory prompts, tend to be more accessible. Gesture-based systems, like those enabled by Kinect, can reduce the need for handheld controllers and offer a more intuitive form of engagement. This aligns with principles from both TAM and behavior change theory, particularly in how user-friendly systems can reinforce positive behavior through immediate feedback and perceived competence. Given the internet connectivity disparities identified in Table 6, particularly in rural or low-resource settings, developers should build an offline mode as a core failsafe rather than an optional feature: core functions such as exercise instructions, activity logging, and safety alerts should remain usable without an active connection, with data queued locally and synchronized automatically once connectivity is restored.

Implementing technology-assisted physical activity interventions for older adults requires careful consideration of numerous factors, including acceptability, evaluation, accuracy and reliability, user perceptions, safety features, and overall acceptance.

#### 6.1.1 Accessibility and Usability

The simplicity of the user interface (UI) is paramount for ensuring accessibility. Research shows that older adults often struggle with complex interface layouts, a challenge that can be exacerbated by reduced visual acuity and cognitive impairments (69). For instance, an evaluation of smartphone launchers explicitly designed for older adults found that interfaces with larger buttons and reduced scrolling significantly enhance usability for this population (70). Such design considerations lead to improved navigation and faster task completion rates, fostering greater engagement with health-promoting technologies (71).

#### 6.1.2 Protection and Safety

Safety is a crucial concern when designing technology to promote physical activity among older adults. Technology itself should be designed with safety features that minimize risks when engaging in physical exercise. For example, systems can be designed to incorporate safety alerts or reminders for users based on their physical capabilities and health status. This may include prompting users to take breaks during exercise or issuing warnings when detected activity levels exceed recommended thresholds, thereby reducing the risk of injury (72, 73). Additionally, ensuring a safe physical environment is essential. Technologies should enable monitoring of the surroundings to adapt to the user's environment. For instance, innovative environments can detect hazards (such as floor obstacles) and alert older adults to potential dangers before they engage in physical activities (72, 74). Integration of assistive technologies that monitor vital signs or provide real-time feedback during physical activities can further enhance safety. Wearable devices or smartphone applications can track physiological parameters, such as heart rate and movement patterns, to provide users with crucial feedback that can inform their activities and ensure their safety (see Table 2). This proactive approach to safety not only protects older adults but also encourages them to remain active without fear of adverse physical consequences.

#### 6.1.3 Acceptability and User Perception

Acceptability of technology-assisted interventions is critical. Studies show that older adults are more likely to embrace technologies that address their needs, enhancing their autonomy and well-being (2, 75). Research has shown that when technologies are intuitive and user-friendly, older adults tend to report greater satisfaction and a higher willingness to engage with them (76). User perception is further influenced by prior technology experiences and the perceived usefulness of the intervention (75, 77). For instance, completing usability assessments, such as the Quebec User Evaluation of Satisfaction with Assistive Technology (QUEST), can provide insights into how older adults perceive the effectiveness of these devices (76). Additionally, addressing the digital divide by considering the varying levels of technological literacy among older adults can significantly influence the acceptance of technology (78).

#### 6.1.4 Accuracy

The accuracy of data collected through wearable devices or applications is crucial for adjusting interventions based on users’ real-time performance (76). Research emphasizes the need to integrate validated metrics into activity trackers to provide reliable feedback, thereby enhancing users’ adherence to activity goals (79). The use of evidence-based approaches to evaluate the technology's effectiveness can help tailor it to meet the specific needs of older adults (2, 80).

### 6.2 What aspects should researchers consider?

Investigating technology-assisted PA interventions for older adults requires a balance between innovation and practical validation. Researchers should use multidimensional evaluation approaches that consider intervention outcomes, implementation, and scalability. The updated JBI Model of Evidence-Based Healthcare provides an overarching evaluation perspective through its FAME dimensions—feasibility, appropriateness, meaningfulness, and effectiveness (81). Researchers should also consider real-world impact through implementation science and evaluate cost-effectiveness to support scalability. In this regard, using hybrid RCT designs allows simultaneous testing of effectiveness and implementation, helping identify key barriers and facilitators, from user health and digital literacy to system readiness, crucial for successful real-world adoption.

Recruiting older adults into technology-assisted PA programs faces several challenges, including limited access to technology, insufficient skills, and a lack of knowledge, which hinder inclusive participation and adherence. Also, vulnerable groups, such as individuals with chronic illnesses or those with disadvantaged socio-economic status, are particularly affected, further exacerbating their barriers to engaging in PA programs (82). A recent consensus statement provides considerations and recommendations for recruiting older adults in physical activity studies (4). To improve the accessibility and impact of the intervention, health literacy (knowledge of the intervention's relevance) and digital literacy (competence with the technology) should be addressed during recruitment and the intervention phases (4, 83). Special care should be taken to include vulnerable groups, with content, style, and attitude underlining the communication strategy, emphasizing inclusiveness, adding value beyond the PA itself, and highlighting the role of key influencers (84).

No single instrument captures all relevant dimensions of technology-assisted PA interventions, including usability, acceptability, satisfaction, engagement, and implementation outcomes. Researchers should therefore select validated measures according to the specific constructs being evaluated and, where appropriate, combine self-report measures with objective use data and qualitative feedback (85).

The updated JBI Model of Evidence-Based Healthcare identifies four dimensions relevant to the evaluation of healthcare practices: feasibility, appropriateness, meaningfulness, and effectiveness (FAME) (81). Applied to technology-assisted PA interventions, feasibility concerns whether an intervention can be practically delivered in the intended setting given the available resources, costs, technical infrastructure, professional competencies, and user capacity. Appropriateness concerns its fit with the target population, clinical and cultural context, and delivery setting. Meaningfulness concerns how older adults experience the intervention and whether it aligns with their values, preferences, and perceived needs. Effectiveness concerns whether the intervention achieves its intended behavioral and health outcomes without unacceptable harm. These dimensions provide an overarching evaluation perspective rather than a single measurement instrument and should therefore be assessed using appropriate quantitative and qualitative measures.

The Telemedicine Satisfaction Questionnaire [TSQ (86);] may complement this evaluation when an intervention includes a substantial telemedicine component, but it should not be treated as a generic measure for all technology-assisted PA interventions.

Researchers should carefully evaluate how their intervention supports user engagement, as this may influence both technology-use adherence and activity adherence and, ultimately, intervention outcomes. From a research perspective, this means designing and evaluating intervention features that sustain interaction with the technology, such as gamification, adaptive feedback, reminders, and social components, while also facilitating continued participation in physical activity itself. Researchers should document how each design element contributes to short- and long-term engagement, and assess both forms of adherence through objective (e.g., device logs) and subjective (e.g., self-reports, satisfaction scales) measures. Integrating these insights into iterative development cycles may help ensure that the technology remains engaging, effective, and acceptable across diverse user groups.

### 6.3 What aspects should clinicians consider?

For clinicians implementing technology-assisted physical activity (PA) programs for older adults, a structured, clinically grounded assessment process is essential to ensure safety, feasibility, and therapeutic relevance. Mois et al. (87) outlined best practices for implementing technology-based interventions, including adaptive training programs, ongoing technical support, and personalized feedback mechanisms. Table 1 provides a practical overview of key assessment domains across three phases: pre-intervention, peri-intervention, and post-intervention. It outlines the clinical aims for each domain and offers concrete examples of how they can be operationalized in routine practice. By systematically addressing medical safety, functional capacity, motivation, digital readiness, ongoing monitoring, and post-intervention outcomes, clinicians can make informed decisions about eligibility, supervision level, personalization, and long-term follow-up, thereby enhancing both patient safety and intervention effectiveness.

## 7 Discussion

This paper synthesizes current evidence and provides actionable guidance for developing, researching, and implementing technology-assisted physical activity interventions for older adults. Our insights reveal that successful digital health solutions require the deliberate integration of clinical evidence, behavioral theory, technical rigor, and user-centered design principles.

### 7.1 Aligning digital delivery with evidence-based exercise practice

Technology-assisted interventions should not “add PA” as an afterthought; they should operationalize established recommendations and enable high-quality exercise prescription and progression over time. In practice, this means that technology-assisted interventions must operationalize established PA guidelines (7, 19) through digital features that support adherence to FITT-VP principles. This requirement is explicitly addressed in the PETIO guideline (100), which extends CONSORT reporting standards by integrating exercise-specific and age-related dimensions, including the use of technology, digital delivery modalities, tailoring, adherence monitoring, and contextual factors.

The dose-response relationship between PA and health outcomes necessitates sustained engagement with both the prescribed activities and the delivering technology—a dual adherence challenge unique to digital interventions. Digital tools offer scalable delivery mechanisms but must be designed with fidelity to these guidelines rather than technological novelty.

To illustrate how these principles translate into practice, Table 5 presents a worked example for a hypothetical app-delivered walking program for a community-dwelling older adult at low fall risk. Rather than treating FITT-VP as a checklist to be cited, each parameter is paired with a concrete prescribed value and with the two dual-adherence metrics (Section 3) that would allow a researcher or clinician to determine, from routinely collected app and wearable data, whether a lack of program effect reflects insufficient activity dosage, insufficient technology engagement, or both.

This same template can be adapted to other modalities (e.g., resistance training, balance exercise) by substituting the relevant intensity metric (e.g., load or repetitions-in-reserve instead of heart-rate reserve) while retaining the parallel activity-component and technology-engagement columns. The metrics are illustrative and should be defined according to the intended function and prescribed use of the technology; greater interaction should not automatically be interpreted as better adherence.

### 7.2 Technical foundations and practical constraints

Technical considerations, such as accuracy, reliability, connectivity, security, and interoperability, directly determine the feasibility and effectiveness of interventions. Critical infrastructure challenges include internet connectivity disparities in rural areas, device charging compliance, and the necessity of real-time technical support, particularly for older users with limited digital literacy. Privacy-preserving artificial intelligence approaches (federated learning, split learning) enable personalized adaptation while protecting sensitive health data, though computational requirements may limit home-based implementation. Healthcare system integration remains aspirational; few platforms achieve seamless interoperability with electronic health records, limiting clinical utility.

### 7.3 Behavioral science as the foundation for engagement

Technology acceptance models explain adoption intentions but provide limited insight into sustained engagement—the ultimate determinant of intervention effectiveness. Our synthesis reveals that acceptance is necessary but insufficient: long-term adherence depends on interventions that successfully target behavioral and motivational mechanisms. Established behavior change theories (Table 3) converge on core mechanisms: self-efficacy enhancement, autonomous motivation, goal-setting, self-monitoring, feedback, and social support. The Affective-Reflective Theory offers critical insight into exergaming contexts, where hedonic enjoyment drives continued participation beyond rational deliberation. Effective technology-assisted PA programs operationalize these theoretical constructs through specific behavior change techniques embedded in digital features.

The key challenge is moving beyond nominal reference to theory toward systematic application. Many digital health studies cite behavioral frameworks without demonstrating how specific application features map onto theoretical mechanisms or demonstrating fidelity to behavior change technique taxonomies. Future research must explicitly document this translation from theory to design.

### 7.4 User-centered design as implementation strategy

The co-creative methodologies outlined in Table 7 represent more than ethical best practice—they are strategic necessities for developing acceptable, usable solutions. Older adults’ heterogeneity in physical capacity, cognitive function, sensory abilities, and technology experience demands iterative co-design cycles across all technology readiness levels. Participatory Action Research and Experience-Based Co-Design position older adults as co-researchers rather than passive subjects, capturing lived experience that reveals barriers invisible to developers.

Usability principles for older adults are well established: large fonts, clear iconography, minimal scrolling, and multimodal feedback, but implementation gaps persist. The tension between feature-rich applications and simplicity often resolves in favor of complexity, alienating the target population. The optimal solution varies by individual, reinforcing the need for adaptable, customizable designs rather than one-size-fits-all products.

### 7.5 Stakeholder-specific implications

This paper differentiates guidance for three key stakeholder groups. Developers should prioritize: (1) accessibility standards meeting Web Content Accessibility Guidelines criteria; (2) embedded behavior change techniques grounded in explicit theoretical frameworks; (3) technical performance validation in older adult samples; (4) privacy-by-design architecture with transparent data governance; and (5) co-creation partnerships throughout development cycles.

Researchers must: (1) apply comprehensive pre-intervention assessments spanning health status, functional capacity, digital literacy, and technology acceptance using validated instruments (Table 1); (2) document recruitment strategies addressing digital divide barriers and vulnerable population inclusion; (3) evaluate dual adherence, activity completion and technology engagement, using objective device logs and subjective satisfaction and usability measures; (4) assess safety through adverse event monitoring; and (5) use the FAME dimensions - feasibility, appropriateness, meaningfulness, and effectiveness - as an overarching evaluation perspective.

Clinicians should: (1) screen patients for PA readiness and technology capacity; (2) provide individualized orientation sessions that build self-efficacy; (3) establish technical support pathways leveraging family networks and professional resources; (4) monitor adherence and adapt prescriptions based on objective data; and (5) evaluate outcomes using functional measures alongside technology usability assessments.

### 7.6 Addressing equity and ageism

Digital health interventions risk exacerbating health inequities if access barriers, cost constraints, and algorithmic biases remain unaddressed. Socio-economic disparities in device ownership and internet connectivity systematically exclude disadvantaged populations from technology-assisted programs. The digital divide extends beyond access to digital literacy—many older adults possess devices but lack the skills or confidence to use health applications effectively. Targeted support addressing both health literacy (understanding the relevance of the intervention) and digital literacy (operating the technology) is essential during recruitment and intervention phases.

Ageism in artificial intelligence represents a particularly insidious challenge. Machine learning algorithms trained predominantly on younger populations may underperform or produce biased risk predictions in older adults owing to shifts in the distribution of physiological data patterns. The WHO (101) policy brief on ageism in AI for health emphasizes age-inclusive data collection and diverse development teams as mitigation strategies. Transparent algorithmic accountability and routine bias audits should become standard practice.

## 8 Limitations and future directions

This position statement synthesizes existing evidence while acknowledging several limitations. First, the rapid pace of technological innovation may render specific device recommendations outdated; our focus on underlying principles rather than products aims to provide enduring guidance. Second, most included evidence derives from high-income countries with established digital infrastructure; applicability to low-resource settings requires contextualization. Third, heterogeneity in study designs, outcome measures, and reporting quality limits the ability to draw definitive conclusions about intervention effectiveness.

Future research priorities include: (1) hybrid effectiveness-implementation trial designs evaluating real-world feasibility alongside efficacy; (2) long-term follow-up studies assessing sustained behavior change beyond immediate intervention periods; (3) cost-effectiveness analyses informing scalability decisions; (4) comparative effectiveness research identifying optimal technology modalities for specific outcomes and populations; and (5) mechanistic studies elucidating pathways through which digital features influence behavioral and health outcomes.

## 9 Conclusion

Technology-assisted physical activity interventions hold substantial promise for addressing population aging challenges, but realizing this potential requires moving beyond a technology-push paradigm towards an evidence-driven, user-centered approach. By integrating clinical guidelines, technical standards, behavioral science, and co-creative design, stakeholders can develop digital health solutions that are not only innovative but also acceptable, effective, and equitable. The guidance provided herein offers a practical roadmap for researchers, developers, and clinicians committed to translating digital innovation into meaningful health improvements for older adults.
